# Study on Bond-Slip Behavior between Seawater Sea-Sand Concrete and Carbon Fiber-Reinforced Polymer (CFRP) Bars with Different Surface Shapes

**DOI:** 10.3390/polym14132689

**Published:** 2022-06-30

**Authors:** Jing Gao, Penghai Xu, Lingyun Fan, Giovanni Pietro Terrasi

**Affiliations:** 1 Department of Civil Engineering, Xiamen University, Daxue Road 182, Xiamen 361005, China; 25320211152338@stu.xmu.edu.cn (P.X.); fly950920@163.com (L.F.); 2 Mechanical Systems Engineering Laboratory, Empa, Swiss Federal Laboratories for Materials Science and Technology, Ueberlandstrasse 129, 8600 Duebendorf, Switzerland; giovanni.terrasi@empa.ch

**Keywords:** CFRP bar, seawater sea-sand concrete cube, bond-slip behavior, pull-out test

## Abstract

The application of CFRP bar and seawater sea-sand concrete (SSSC) in construction can overcome the shortcomings in conventional reinforced concrete, such as corrosion induced by carbonation and chloride ingress. In this study, the bond-slip behavior between an SSSC cube and CFRP bar has been investigated, and different CFRP bar surface shapes have been considered. A total of 27 specimens (9 groups) were fabricated for a pull-out test, where three types of CFRP bar with different surface shapes were used: smooth regular bars, double-wrapped bars and ribbed bars. Bond strength, bond-slip curve, and failure mode have been presented and discussed. FE models have been constructed and validated by experimental results. The effect of concrete compressive strength and relative area of ribs on bond strength has been studied through numerical simulations. It is found that the bond strength increased with concrete compressive strength, and the ribbed bar had significantly higher bond strength than the smooth regular bar. Pull-out failure was observed when the cover-depth-to-bar-diameter ratio was no less than 4 and, otherwise, splitting failure occurred. In addition, a simple formula has been proposed to approximately evaluate the bond strength between an SSSC cube and CFRP bar and validated by experimental results, and analytical expressions for different bond-slip curves have also been developed.

## 1. Introduction

Seawater sea-sand concrete (SSSC) composed of sea-sand aggregate and seawater avoids material shortage in ocean construction and shows high economic value, attracting a lot of attention [1,2]. Li et al. [3] investigated the long-term performance of seawater sea sand and coral sand concrete. Saleh et al. [4] developed ultra-high-performance concrete by using seawater and sea sand. Qiao et al. [5] investigated the mechanical properties including elastic modulus, failure mode, strain–stress curve, etc. of SSSC subjected to variations of temperature. However, chloride ingress in SSSC causes and accelerates corrosion of steel rebar, leading to early deterioration of concrete and eventually to structural failure [6,7,8].

Therefore, in order to prevent the reinforced bars from being corroded by chloride ions in seawater and sea sand, the fiber-reinforced polymer (FRP) bar, which has great corrosion resistance and high tensile strength, is used instead of steel reinforcing bars to improve the concrete durability [9,10,11,12,13]. Mohamed et al. [14] investigated both durability and mechanical properties of concrete reinforced by basalt fiber-reinforced polymer (BFRP) bars and indicated that the durability was influenced by the resin type and bar size. Yu et al. [15] studied the effect of water and alkaline solution on durability of concrete reinforced by carbon–glass hybrid FRP bar. Dong et al. [16] conducted a life cycle assessment case study to show the environmental impact on seawater sea-sand concrete beam reinforced with glass fiber-reinforced polymer (GFRP) bars and CFRP bars.

So far, FRP bars have been widely used not only in marine engineering, but also in many other special constructions, due to the excellent mechanical properties and durability. The structural behavior of concrete reinforced by FRP bars is important for real applications, especially for flexural strength and shear strength. Jiang et al. [17] investigated the performance improvement of SSSC beams reinforced with FRP bars in serviceability limit state. Karayannis et al. [18] designed an experiment to investigate the behavior of slender concrete beams reinforced with CFRP bars including deflection, failure mode, and cracking pattern, etc. Ogrodowska et al. [19] presented the impact of nanomodification, hybridization and temperature on the shear strength of BFRP bars. Pang et al. [20] investigated the flexural behavior of two-span continuous CFRP bar-reinforced concrete beams. Roudsari et al. [21] proposed an optimization method to improve the structural performance of FRP bars-reinforced concrete columns by using nonlinear finite element (FE) model. Gu et al. [22] designed and conducted an experimental study to investigate the flexural properties of hybrid FRP bars-reinforced concrete beams. Guo et al. [23] developed a method based on accumulative damage model to predict the fatigue life of CFRP bar-reinforced concrete beams. Kim et al. [24] evaluated the structural performance of concrete structural component reinforced with FRP bars by different bars. Bakar et al. [25] reviewed the flexural strength of CFRP bar-reinforced concrete beams and indicated that both ACI 440.1R-15 and CSA S806-12 underestimate the ultimate flexural moment capacity of CFRP-reinforced concrete beams.

Besides structural behavior, bond performance between FRP bar and concrete is also important. Due to the difference in the chemical and mechanical properties between FRP and steel bars, the bond performance between FRP bar and concrete cannot be directly calculated using the traditional steel–concrete bond-slip model [26,27,28]. Yin et al. [29] investigated the bond performance between different types of FRP bars and all-coral aggregate seawater concrete by pull-out test, and showed that pull-out failure was mainly observed in specimens with smooth round bars and deformed CFRP bars and splitting failure was usually found in specimens with deformed BFRP and GFRP bars. In fact, the bond performance between FRP bars and concrete is affected by several factors such as material properties, and cover depth, etc. Many experimental and numerical studies have been conducted to investigate the factors influencing the bonding performance between FRP bars and concrete [30,31,32,33,34,35]. In particular, Caro et al. [36] analyzed the effect of FRP bar diameter, concrete strength, and bond length on the bonding performance between FRP bars epoxy-bonded into concrete. Veljkovic et al. [37] studied the influence of protective layer thickness on the bond performance between GFRP bars and concrete. They showed that the bond strength of ribbed GFRP bars was greater and the slip value was lower when the protective layer thickness was small, because the failure mode changed to splitting failure rather than pull-out failure. Jeong et al. [38] investigated the fatigue behavior of partially bonded CFRP bar-reinforced concrete beams subjected to cyclic load. Zhao et al. [39] carried out an experimental study to evaluate the bond behavior between CFRP bars, GFRP bars, steel strands and concrete, and pointed out the bond strength retention rate of GFRP bars was the highest, while that of steel strand was the lowest. Gong et al. [40] investigated the bond performance between CFRP bars and the cracked concrete interface by conducting experiments. Zhou et al. [41] investigated the bond performance between FRP bars and advanced sustainable concrete where seawater, sea sand, and recycled aggregates were used.

However, there is limited research on the bond behavior between CFRP bars and SSSC, due to the unique characteristics of CFRP bars, especially for different surface shapes of CFRP bars. Therefore, this paper conducted the pull-out tests on an SSSC cube with CFRP bar to investigate the bond performance and the main factors influencing the bonding performance, such as concrete compressive strength and relative area of ribs of a ribbed CFRP bar. In addition, refined FE models were constructed and validated by experimental results, based on which a simple formula was proposed to evaluate the bond strength approximately, and analytical expressions for different bond-slip curves were developed as well.

## 2. Experimental Program

The properties of the adopted SSSC cube and CFRP bar, and the test procedures and results, are presented in this section.

### 2.1. Material Properties

**Figure 1:**
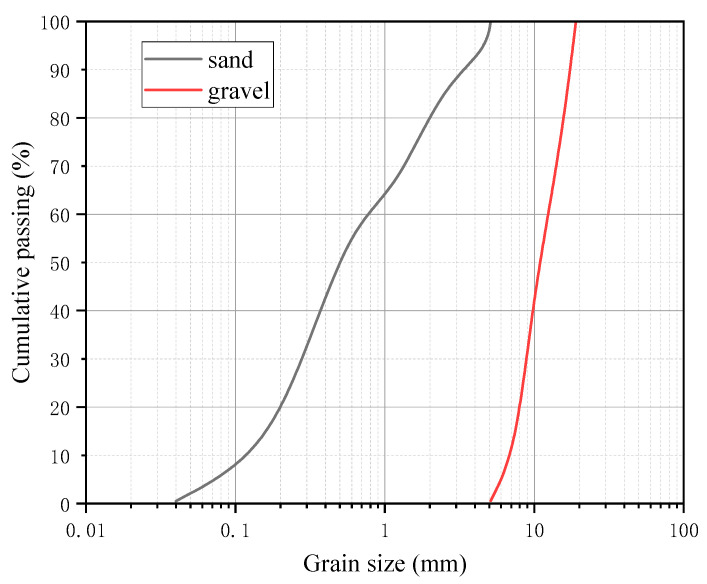
Grain size distribution of sand and gravel aggregate.

The seawater and sea sand used in this study were all produced naturally in Xiamen, China. The cement used in this study was ordinary Portland cement with compressive strength of 42.5 MPa after 28 days. The grain size distribution of sand and gravel aggregate can be seen in Figure 1. High performance polycarboxylate superplasticizer with a water reducing rate of 20% was used to ensure the proper workability of concrete. Class I fly ash was used as the admixture and the content was 20%. Three grades of concrete—C30, C40, and C50—were adopted in this study, and the details of concrete mixture can be seen in Table 1, where “W/C” ratio means the water-to-cement ratio.

**Figure 2:**
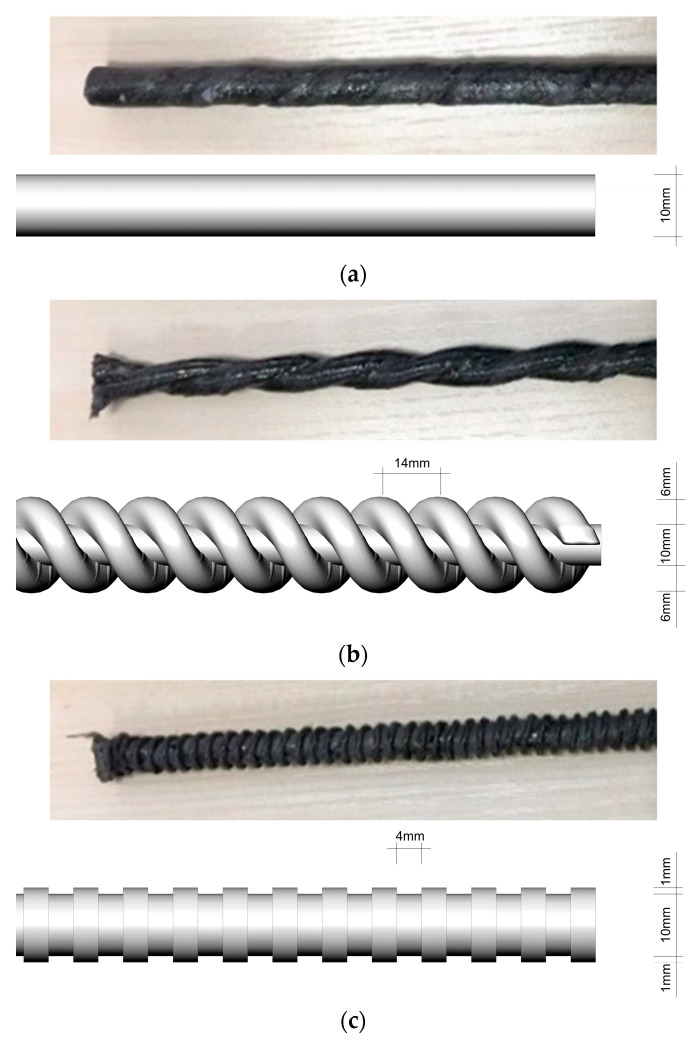
CFRP bars with different surface shapes: (**a**) smooth regular bars; (**b**) double-wrapped bars; (**c**) ribbed bars.

High-adhesion carbon fiber prestressed bars were used as the CFRP bars, which had a diameter of 10 mm in this study. Three types of CFRP bars with different surface shapes, namely, smooth regular bars, double-wrapped bars, and ribbed bars, were selected. Figure 2 shows the three types of CFRP bars with different surface shapes, and Table 2 presents their elastic modulus and tensile strength, which were determined by tensile testing.

### 2.2. Specimens

**Figure 3:**
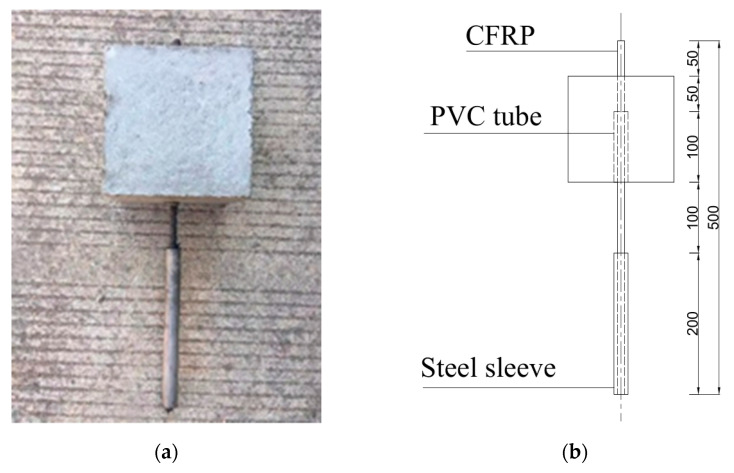
Pull-out specimen: (**a**) photograph; (**b**) illustration.

Each specimen consisted of a CFRP bar, a PVC tube, a steel tube, and a concrete cube, as shown in Figure 3. The concrete cube for each specimen had the dimensions of 150 mm × 150 mm × 150 mm, followed by ACI 440.3R-12 [42], Chinese national code [43], and previous studies [34]. A steel tube was adhered to the loading end of CFRP bar to protect the bar during pull-out test, while the other end of the CFRP bar was freely exposed. The bond length between CFRP bar and concrete was 50 mm, and a 100 mm-long PVC tube was used to separate CFRP bar and concrete, because the ratio of embedded length to diameter of rebar should be 5 [42,43]. Once the specimens were cast, they were cured at standard conditions for 28 days.

In this study, the identification of specimen is determined as follows: the first letter indicates the CFRP bar type (S for smooth regular bar, D for double-wrapped bar, and R for ribbed bar), the first number indicates the grade of concrete, the second number indicates the ratio of cover depth to diameter of CFRP bar (c/d), and the last number donates the specimen number. For example, “S-50-7-3” designates the third specimen of the smooth regular CFRP bar-reinforced C50 SSSC cube with cover-depth-to-bar-diameter ratio of 7. A total of 27 specimens were cast in 9 groups based on orthogonal test design, which are listed in Table 3. For each group, three specimens were prepared to ensure data reliability.

### 2.3. Pull-Out Test

**Figure 4:**
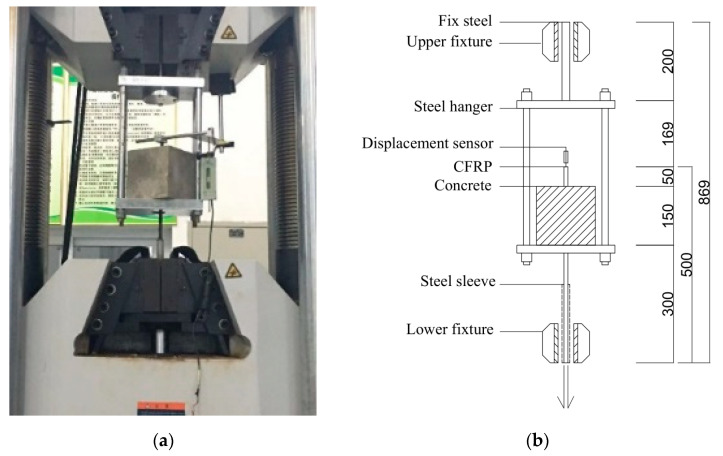
Experimental setup of pull-out test: (**a**) photograph; (**b**) illustration.

Figure 4 illustrates the experimental setup of pull-out test. The specimen was held by a frame consisting of two steel plate and four rods, the upper plate was connected to the test machine, and the lower plate had a hole to allow the CFRP bar to pass through. The relative slip between SSSC cube and CFRP bar were measured by LVDT. A universal testing machine was employed to apply the tensile load under displacement control at 0.5 mm/min.

### 2.4. Test Results

In the pull-out test, since the stress distribution is not uniform, which means that the shear stress along the axial direction ($\tau_{x}$) is not exactly a constant, the average bond stress along the embedded CFRP bar (τ) is therefore adopted:

(1) $$\tau =\frac{\int_{0}^{l}\tau_{x}dx}{l}=\frac{P}{\pi dl}$$

where P is the pull-out load measured by the universal testing machine, d is the diameter of CFRP bar, and l is the length of embedded CFRP bar. The relationship between the average bond stress and the unloaded CFRP bar end slip is used to analyze the bond behavior in this study. The test results have been summarized in Table 4, including the specimen ID, average bond strength, the peak slip related to the maximum load, and the failure mode (P = pull-out failure, S = splitting failure). In particular, the predicted bond strength by ACI 440.1R-06 [44] listed in Table 4 shows that the ACI code is not conservative for the bond strength between SSSC and CFRP bars with three different surface shapes, which indicates that this study is of significance.

#### 2.4.1. Failure Mode

Two typical failure modes were observed during the pull-out tests: CFRP bar pull-out failure and concrete splitting failure.

**Figure 5:**
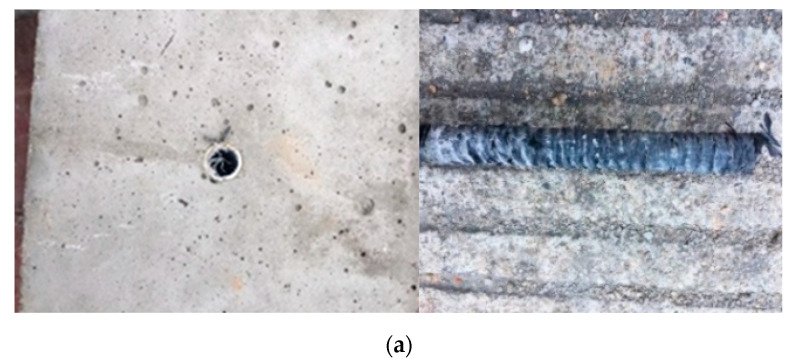

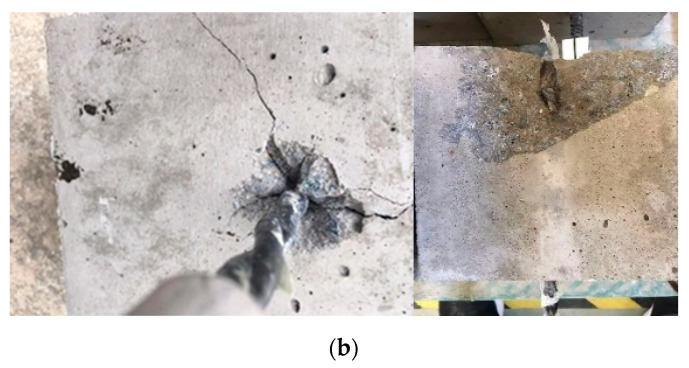
Different types of failure mode: (**a**) CFRP bar pull-out failure; (**b**) concrete splitting failure.

For the CFRP bar pull-out failure, the surface of the concrete did not change significantly except that a small portion of the concrete near the bonding interface spalled off, and the CFRP bar was pulled out without damage, as shown in Figure 5a. Such failure mode occurred at the interface once the pull-out stress exceeded the shear bond strength between CFRP bar and concrete. Generally, when the ratio of cover depth to bar diameter (c/d) was no less than 4, the CFRP bar pull-out failure can be observed, since the surrounding concrete could still provide sufficient confinement to prevent splitting. This failure mode is usually preferred in actual application because it can provide an accurate evaluation of the bonding strength between CFRP bar and concrete.

For the concrete splitting failure, a large portion of the concrete spalled off, since the surrounding concrete can never provide adequate confinement before the pull-out stress exceeded the shear strength between CFRP bar and concrete, as shown in Figure 5b. There was a thin layer of concrete around the bar surface and the interface between CFRP bar and concrete was still undamaged, indicating that the bond strength was not attained. The radical stress due to the pull-out load initiated cracks in the radical direction from the interface, and the concrete confinement was not sufficient to prevent the crack propagation. This failure mode was only observed for smooth regular bar and double-wrapped bar when the ratio of cover depth to bar diameter was 2.

#### 2.4.2. Bond-Slip Curves

**Figure 6:**
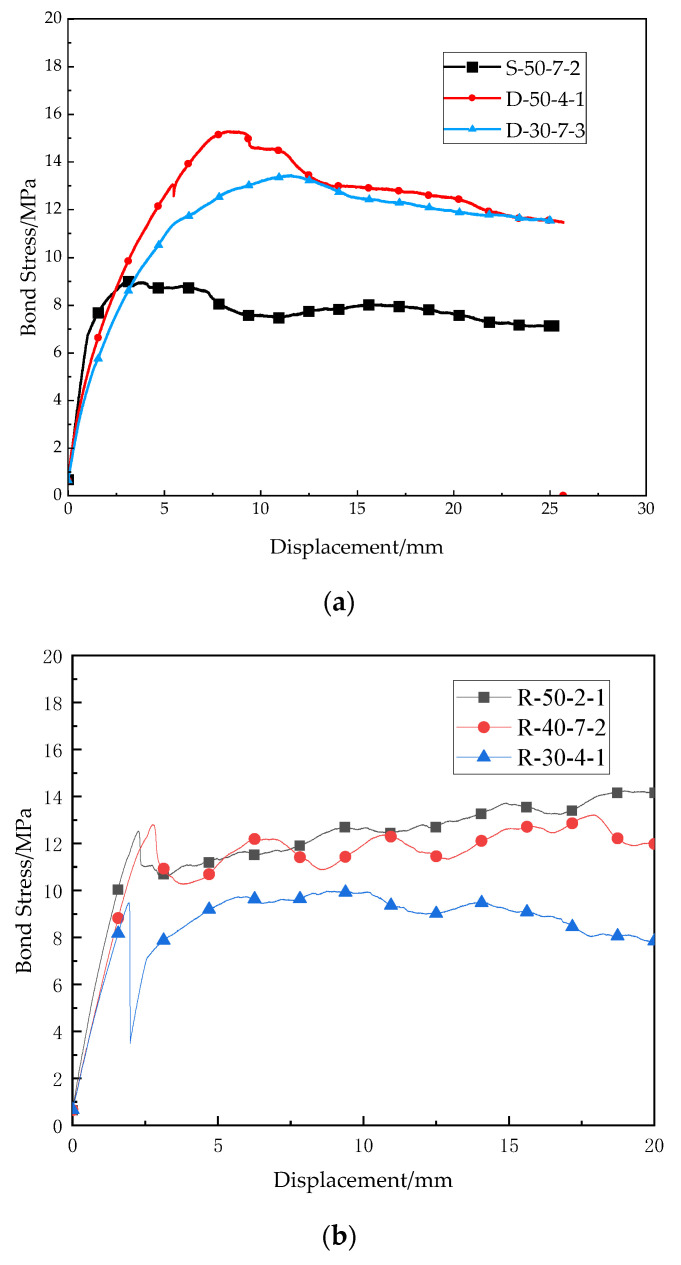

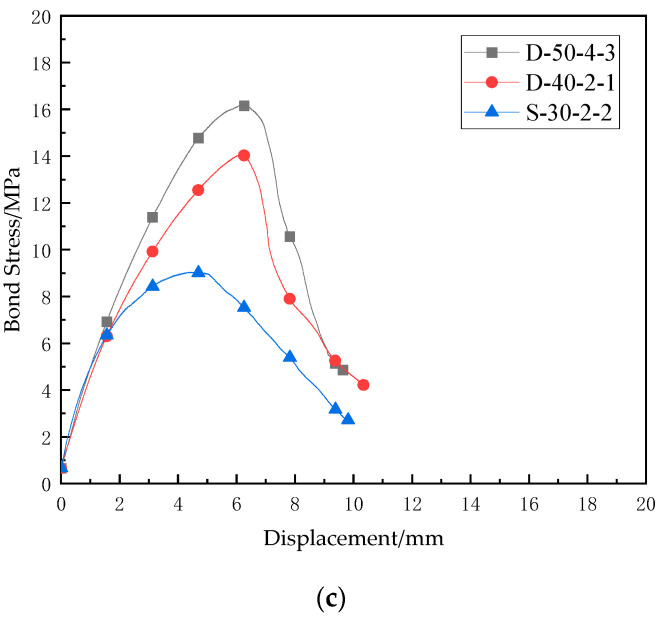
Bond-slip curves of different failure mode (**a**) bar pull-out failure (smooth regular CFRP bar) (**b**) bar pull-out failure (double ribbed CFRP bar) (**c**) concrete splitting failure.

As discussed above, there were two typical failure modes. Each failure mode has its own bond-slip relationship, which can be seen in Figure 6.

Figure 6a shows the bond-slip curve of smooth regular CFRP bar subject to bar pull-out failure (S-50-7-2, D-50-4-1, D-30-7-3). The bond stress increases gradually with the slip until the first peak. After reaching the maximum bond stress (bond strength), the interface between concrete and CFRP bar starts to damage, and the bond stress decreases slightly as the slip continues to increase. With the large increase in slip, the interface is damaged completely, and the bond stress comes from friction rather than bonding interface between concrete and bar. It should be noted that even if the interface was damaged completely in the test, the contact between concrete and bar was still close, and therefore, the bond stress only decreases from the peak value slightly in Figure 6a, which means that the friction can still provide resistance to slip between concrete and CFRP bar.

Figure 6b shows the bond-slip curve of ribbed CFRP bar subjected to bar pull-out failure (R-50-2-1, R-40-7-2, R-30-4-1). Different from the above bond-slip curve, the bond stress increases quickly with the slip at the initial stage, and it can reach the first peak when the slip is only 2 to 3 mm, which means that the bonding between ribbed bar and concrete is great, and the bond stress can achieve a high level with limited deformation of bar. With the increase in slip, the bond stress decreases slightly due to peeling off of some ribs and the corresponding damage of interface. However, the bond stress rises slightly again since the friction between concrete and damaged ribs increases with the increase in slip. Then, the same procedures repeat a few times, and therefore, the bond-slip curve fluctuates periodically.

Figure 6c represents the bond-slip curve of concrete splitting failure (D-50-4-3, D-40-2-1, S-30-2-2). Similarly to the initial stage of the bond-slip curve of CFRP bar pull-out failure, the bond tress rises gradually with the slip until maximum bond stress (bond strength). However, after the peak, the bond stress decreases suddenly because the radial cracks initiate and propagate quickly. Although the interface between concrete and bar is still undamaged, the bar and the nearly surrounding concrete separate from the concrete cube completely, and therefore, the bond-slip curve drops with a great negative gradient after the maximum value.

## 3. Numerical Analysis

FE models simulating the pull-out test of the three types of CFRP bars have been constructed by ABAQUS 2020 and validated by the experimental results. A parametric study has also been conducted to investigate the effect of different factors including concrete compressive strength, CFRP bar diameter, and relative size of rib on bond performance between an SSSC cube and CFRP bar.

### 3.1. FE Models

**Figure 7:**
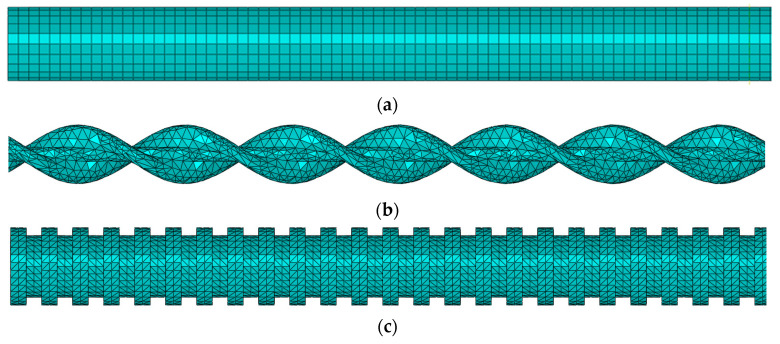
FE models of (**a**) regular bar. (**b**) double-wrapped bar, and (**c**) ribbed bar.

The FE models of three bars with different surface shapes are shown in Figure 7. The diameter for all three bars is 10 mm. The double-wrapped bar has wrapping spacing of 14 mm and the ribbed bar has rib spacing of 4 mm, rib height of 1 mm, and rib width of 2 mm.

**Figure 8:**
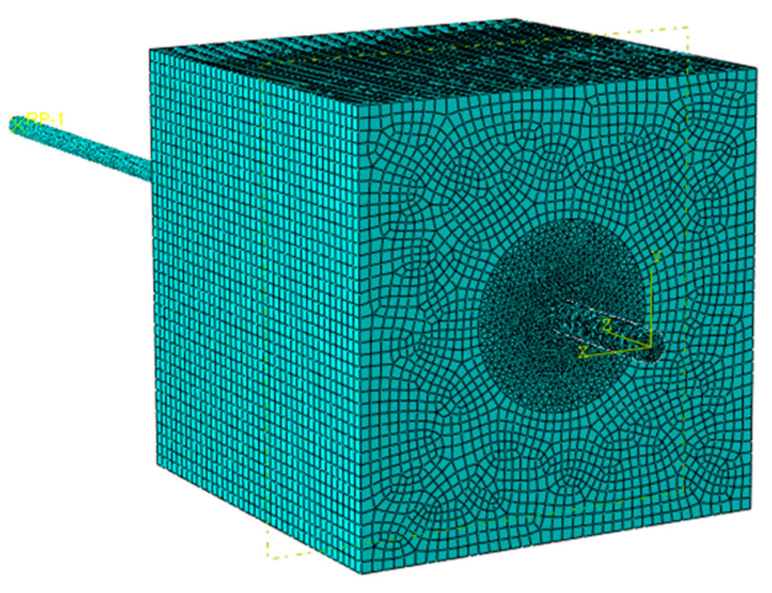
Assembled FE model of CFRP bar and SSSC cube.

The assembled FE model of an SSSC cube and CFRP bar is shown in Figure 8, where the general contact between concrete and CFRP bar was adopted in the FE models. In particular, “Hard Contact” was selected for normal behavior, “Friction” was selected for tangential behavior, and the friction coefficient was set to 0.5. “Finite sliding” was adopted as the sliding formulation to simulate the slip between the concrete and CFRP bar. A gap of 1 mm between concrete and bar was modeled to simulate the PVC pipe. All degrees of freedom of bottom surface of the concrete cube was restrained to simulate fixed boundary conditions. The load was applied to the end of CFRP bar by displacement control, coinciding with the pull-out test. C3D8R element (8-node cuboid element with reduced integration) was used for the concrete cube and regular CFRP bar, and C3D4 element (4-node tetrahedral element) was used for double-wrapped CFRP bar and ribbed CFRP bar. The size of mesh on the CFRP bar was around 1 mm, nearly one tenth of the CFRP bar diameter, to ensure the convergence. In fact, the convergence was checked and it showed that when the mesh size was less than one eighth of the CFRP bar diameter, the results converged. A cylindrical zone with diameter of 60 mm in center of the concrete cube was generated which had the same mesh size with the CFRP bar. The rest of the concrete cube had a mesh size of 4 mm to reduce the total mesh number. The total mesh number of FE models varied from 2 × 10^5^ to 4 × 10^5^.

The concrete damage plasticity (CDP) model was employed as the constitutive model of SSSC to simulate the smeared crack representation, and the detailed parameters can be seen in Table 5, where *f*_b0_/*f*_c0_ is the compression plastic strain ratio and *K* is the invariant stress ratio. It combines damage theory and plasticity theory, and it includes two main failure mechanisms, namely, tensile cracking and compression fractures. The material properties of CFRP bar were obtained from a static test, and they are listed in Table 5 as well. The stress–strain curve of CFRP bar measured in the static test was used as the material property of CFRP bar in the simulation, which can be achieved by keying in measured data in ABAQUS.

### 3.2. Verification of Proposed FE Models

**Figure 9:**
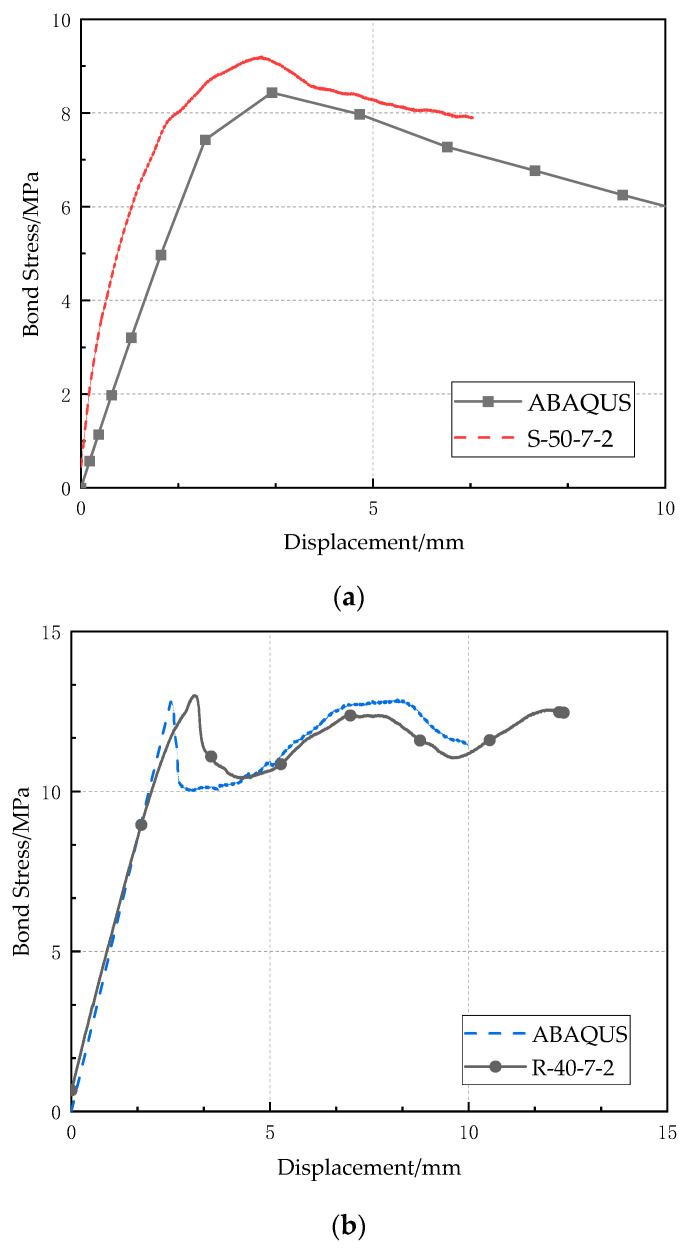
Comparison of bond-slip curves between experimental test and numerical simulation: (**a**) S-50-7-2; (**b**) R-40-7-2.

The FE models have been verified by the experimental results. Figure 9 shows the comparison of bond-slip curves of S-50-7-2 and R-40-7-2, obtained by experimental test and numerical simulation. It should be noted that the surrounding matrix has great stress and strain during the pull-out of the CFRP bar, and therefore, a cylindrical zone of 60 mm diameter having fine meshes had been used in the FE model. The bond stress by ABAQUS in Figure 9 was calculated as the average stress along the CFRP bar in the numerical simulation. Generally, the simulation results match the experimental results well. For specimen S-50-7-2, the maximum bond stress by numerical simulation is quite accurate, although the bond stress increases slightly slower than that measured in the test during the bond stress raising stage. For the specimen R-40-7-2, the FE model can accurately calculate the maximum bond stress and also the following fluctuation in bond-slip curve for the ribbed bar. Other bond-slip curves obtained by numerical simulations agree well with those from the experimental pull-out test, and they are not shown herein for brevity.

### 3.3. Parametric Study

Two factors, concrete compressive strength and relative size of rib, have been considered in this study to investigate the effect on the bond strength by the validated FE models.

#### 3.3.1. Concrete Compressive Strength

**Figure 10:**
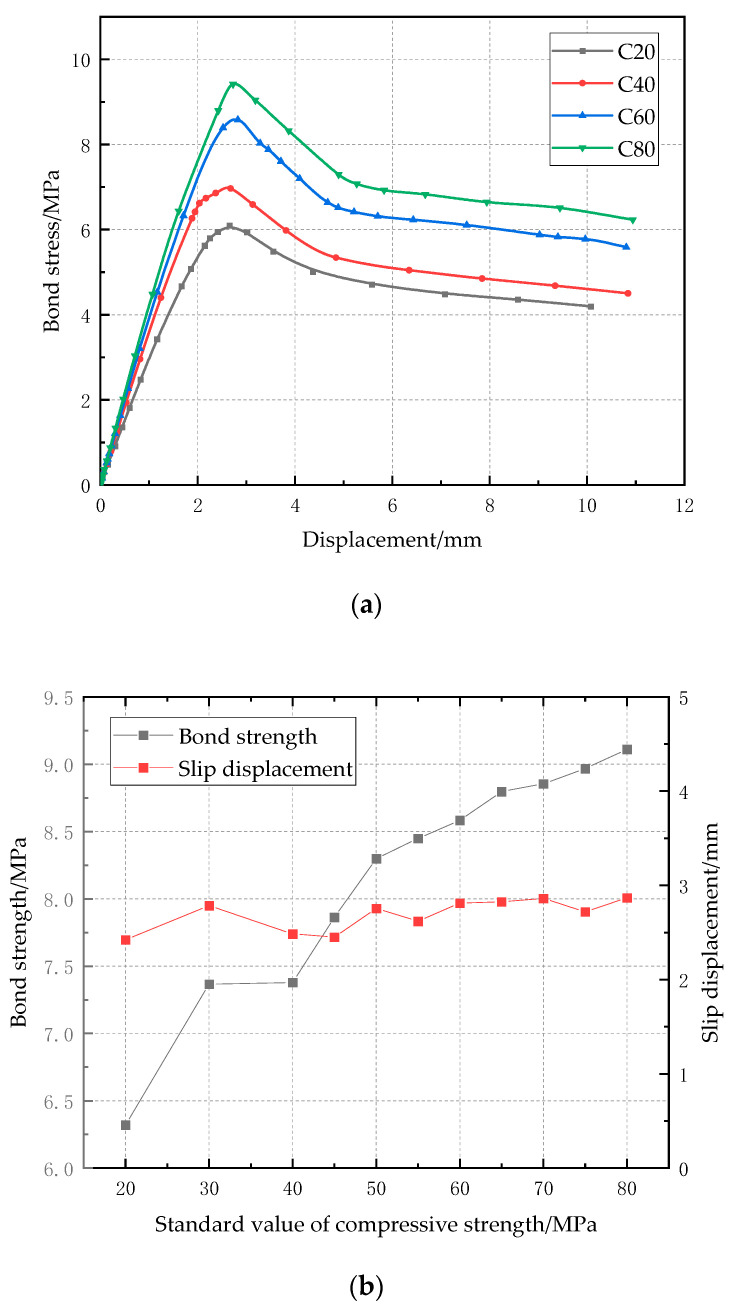
Effect of concrete compressive strength on bond strength: (**a**) bond-clip curves; (**b**) relationship between bond strength/slip displacement and concrete strength.

An FE model of the regular bar with a diameter of 10 mm, ratio of cover depth to bar diameter of 7, was used to investigate the effect of concrete compressive strength on bond strength. Concrete compressive strength was used in the simulation, with 11 different values from 20 MPa to 80 MPa. Figure 10a shows the bond-slip curves with concrete compressive strength of 20 MPa, 40 MPa, 60 MPa, and 80 MPa, respectively. Since the concrete cover depth is sufficient, the CFRP pull-out failure can be observed. Therefore, the bond-slip curves rise to the maximum bond stress and then drop gradually, which coincides with the experimental observation. The relationship between bond strength and concrete strength is shown in Figure 10b. Generally, the bond strength between the concrete and CFRP bar increases as the concrete strength rises, but it should be noted that there is a plateau from 30 MPa to 40 MPa, which means that when the concrete compressive strength increases from 30 MPa to 40 MPa, the bond strength is still about 7.3 MPa without obvious increase. Figure 10b also shows the relationship between slip displacement related to the maximum bond stress and concrete strength, and the former slightly changes in the narrow range from 2 mm to 3 mm, although the latter increases from 20 MPa to 80 MPa, indicating that the concrete compressive strength has negligible influence on the slip displacement related to the maximum bond stress.

#### 3.3.2. Relative Size of Rib

**Figure 11:**
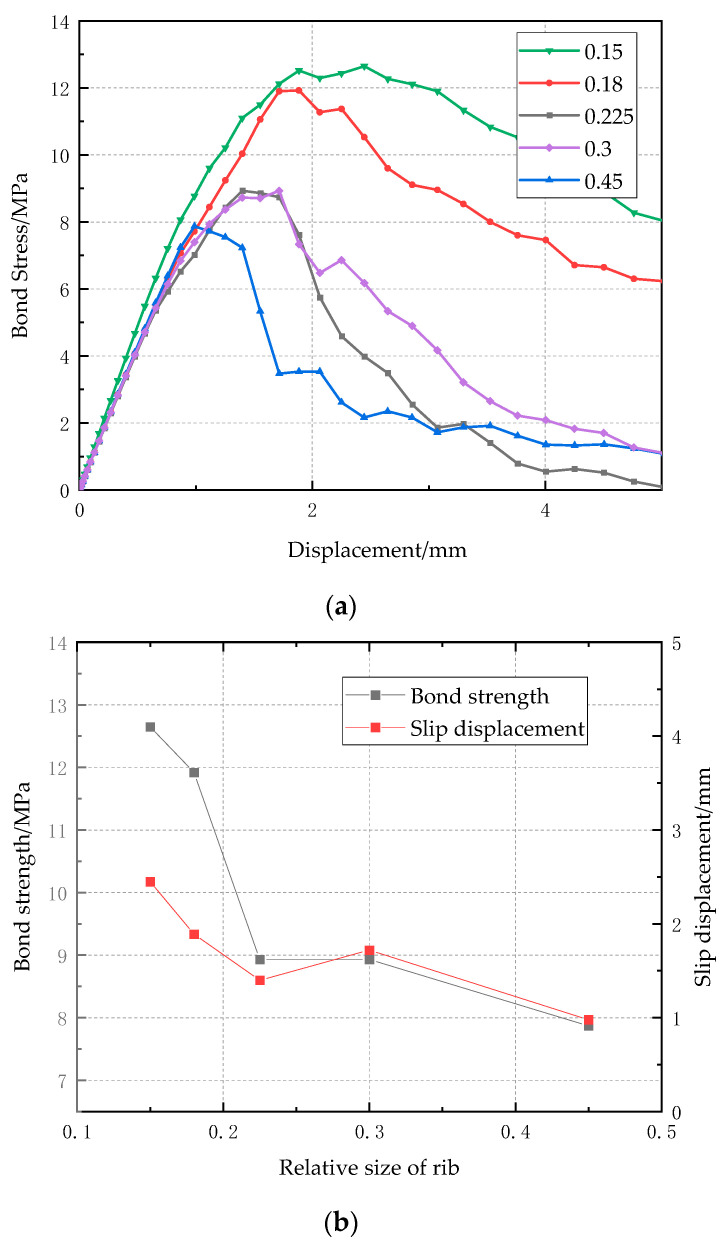
Effect of relative size of rib on bond strength (**a**) bond-clip curves (**b**) relationship between bond strength/slip displacement and relative size of rib.

To investigate the effect of relative size of rib on bond strength, the FE model of the ribbed bar with diameter of 10 mm, ratio of cover depth to bar diameter of 7, and concrete compressive strength of 50 MPa was adopted. The relative size of rib is defined as the ratio of rib area to the cross-sectional area of CFRP bar, and five values, 0.15, 0.18, 0.23, 0.3, and 0.45, have been considered. Figure 11a shows the bond-clip curves with relative size of rib from 0.15 to 0.45. When the relative size of rib is in the range of 0 to 0.18, the CFRP bar pull-out failure occurs, so the bond stress decreases gradually after the maximum bond stress; when the relative size of rib is above 0.2, the failure mode changes to concrete splitting, and the bond stress drops drastically. Figure 11b shows the relationship between bond strength/slip displacement and relative size of rib. It is observed that when the relative size increases from 0 to 0.15, the bond strength increases significantly from 6.8 MPa to 12.5 MPa, and the corresponding slip displacement decrease from 2.5 mm to 1.0 mm, because of the presence of ribs. When the relative size of rib increases to 0.23, the failure mode changes to concrete splitting, and therefore, the bond strength and the corresponding slip displacement drops accordingly.

## 4. Analytical Model

In this section, a simple approximate formula of bond strength has been developed based on the numerical parametric study and validated by the experimental results. In addition, the analytical bond-slip curves for different failure mode has also been proposed.

### 4.1. Bond Strength

It is observed from the numerical study how the three factors influence the bond strength between CFRP bar and SSSC, and therefore, a simple formula of bond strength ($\tau_{u}$) is proposed as

(2) $$\tau_{u}=\beta_{r}\beta_{A}\beta_{c}$$

where

(3) $$\beta_{r}=1.66{\left(\frac{c}{d}\right)}^{0.29}$$

is the factor of cover depth to diameter (c/d) ratio and it is obtained by fitting the curve in Figure 10b by least squares method, and

(4) $$\beta_{A}=\left\{\begin{array}{c} 1.24,\;\mathrm{Concrete}\;\mathrm{spliting}\;\mathrm{failure}\\ 1.80,\;\mathrm{CFRP}\;\mathrm{bar}\;\mathrm{pullout}\;\mathrm{failure} \end{array}\right.$$

is the factor of relative size of rib, and

(5) $$\beta_{c}=2.96f_{c}{}^{0.258}$$

is the factor of concrete compressive strength ($f_{c}$), and it is also obtained by fitting the curve in Figure 9b by the least squares method.

**Figure 12:**
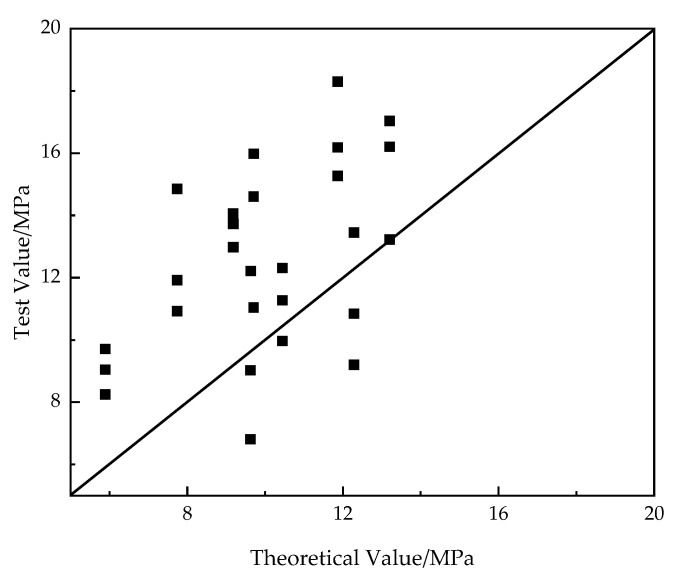
Comparison of bond strength calculated by the proposed formula and measured in the pull-out test.

Figure 12 shows the comparison of bond strength calculated by the proposed formula and measured in the pull-out test. It is observed the formula can predict the bond strength well and conservatively, regardless of the type of CFRP bars. Therefore, the proposed expression of bond strength is applicable for all these types of CFRP bars.

### 4.2. Bond-Slip Curves

**Figure 13:**
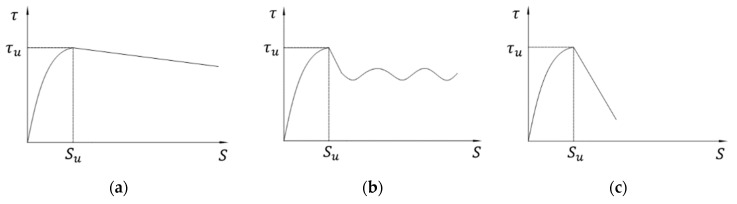
Typical bond-slip curves: (**a**) regular bar and double-wrapped bar subject to bar pull-out failure; (**b**) ribbed bar subject to bar pull-out failure; (**c**) regular bar and double-wrapped bar subject to concrete splitting failure.

The analytical expression of bond-slip curves for the two failure modes have been proposed herein. Figure 13 shows the three typical bond-slip curves observed in the pull-out test. Generally, there are two parts in the bond-slip curve: the rising part where the chemical adhesive force plays a major role, and the sliding part where the bonding force is mainly due to friction.

For the regular bar and double-wrapped bar, the Malvar model [45] and linear model have been used for the rising part and sliding part, respectively, regardless of failure mode:

(6) $$\frac{\tau }{\tau_{m}}=\left\{\begin{array}{c} \frac{F\left(\frac{s}{s_{m}}\right)+\left(G-1\right){\left(s/s_{m}\right)}^{2}}{1+\left(F-2\right)\left(\frac{s}{s_{m}}\right)+G{\left(s/s_{m}\right)}^{2}},0\leq s\leq s_{m}\\ 1-k\left(\frac{s}{s_{m}}-1\right),s>s_{m} \end{array}\right.$$

where s is the slip and $s_{m}$ is the slip displacement corresponding to the maximum bond stress, while F, G, and k are unknown parameters to be determined by experimental results.

For the ribbed bar, after the short sliding part from $s_{m}$ to $s_{w}$, there is a fluctuating part due to the presence of ribs. Therefore, BPE model [46] and linear model have been adopted for the rising part and sliding part, respectively, and a fluctuation function has been selected for the fluctuating part:

(7) $$\tau =\left\{\begin{array}{c} {\left(s/s_{m}\right)}^{a},0\leq s\leq s_{m}\\ \frac{(\tau_{w}-\tau_{m})\;s+\tau_{m}s_{w}-\tau_{w}s_{m}}{s_{w}-s_{m}},s_{m}\leq s\leq s_{w}\\ \tau_{w}-\gamma \left[e^{-\varphi \omega \left(s-s_{w}\right)}\cos\left(s-s_{w}\right)-1\right]\\ +\rho \left(e^{-\varphi \omega \left(s-s_{w}\right)}-1\right),s>s_{w} \end{array}\right.$$

where a, γ, φ, ω, and ρ are unknown parameters to be determined by experimental results.

**Figure 14:**
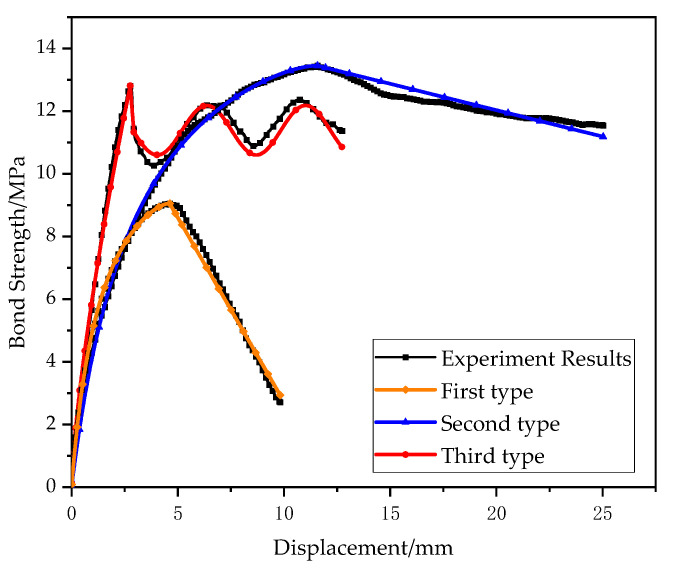
Comparison of fitted bond-slip curves and test results.

Figure 14 shows the fitted bond-slip curves of S-30-2-4, D-30-7-4, and R-40-7-2 for illustration, and Table 6 lists the values of unknown parameters determined by the test results. It is observed that the fitted bond-slip curves and the test results have a great agreement, indicating that the proposed analytical expressions can depict the characteristics of these three typical bond-slip curves well.

## 5. Conclusions

In this study, the bond-slip behavior between an SSSC cube and CFRP bar with different surface shapes has been investigated. A total of 27 specimens (9 groups) were fabricated for the pull-out test, where three different CFRP bar surface shapes were used, namely, smooth regular bars, double-wrapped bars and ribbed bars. Accordingly, bond strength, bond-slip curve, and failure mode have been presented and discussed. FE models of specimens have also been constructed and validated by the experimental results. The effect of concrete compressive strength and relative area of ribs on bond strength has been studied through numerical simulations. In addition, a simple formula has been proposed to approximately evaluate the bond strength between SSSC cube and CFRP bar and validated by test results. The proposed formula is more accurate than the provisions in ACI because the latter is more conservative. Analytical expressions for different bond-slip curves have also been developed. The main findings and conclusions are listed as follows:

1. In the pull-out test, the bond strength was highly dependent on the concrete compressive strength and the surface type of CFRP bars. The bond strength was higher if the concrete compressive strength value was larger, and the ribbed bar had significantly higher bond strength than the regular bar, which were further validated in the numerical simulations. From the numerical simulations, it is further observed that the increasing rate of bond strength became slower with the increase in concrete compressive stress.
2. There were two failure modes observed during the pull-out test mainly depending on the ratio of cover depth to bar diameter—CFRP bar pull-out failure and concrete splitting failure. When the ratio of cover depth to bar diameter was no less than 4, the CFRP bar pull-out failure was observed, and otherwise, the concrete splitting failure occurred, because cracks initiated due to pull-out-load-induced radical stress, and the concrete confinement was not sufficient to prevent the crack propagation.
3. The bond-slip curves for different failure mode were different. Generally, the bond stress dropped significantly after the maximum value for the concrete splitting failure and it decreased gradually (regular bars) or fluctuated slowly (ribbed bars) after the maximum value for the CFRP bar pull-out failure.
4. The proposed simple formula based on the parametric study can approximately predict the bond strength, which is more accurate than those in ACI, and the analytical expressions can depict the bond-slip curves for different failure modes and different shapes of CFRP bar.

## Figures and Tables

**Table 1:** Details of concrete mixture.

Grade	W/C Ratio	Seawater (Kg/m^3^)	Coal Ash (Kg/m^3^)	Cement (Kg/m^3^)	Sand Ratio	Sea Sand (Kg/m^3^)	Gravel Aggregate (Kg/m^3^)	Water Reducer (Kg/m^3^)	Total Mass (Kg/m^3^)	Compressive Strength /MPa
C30	0.521	184	70.7	282.79	0.396	756.26	1152.7	3.535	2450	37.5
C40	0.422	184	87.27	349.06	0.366	669	1156.3	4.363	2450	49.0
C50	0.345	184	106.56	426.24	0.344	593.7	1134.2	5.328	2450	58.9

**Table 2:** Mechanical properties of CFRP bars.

Surface Type	Elastic Modulus (GPa)	Tensile Strength (MPa)
Smooth regular bars	180.0	1862.3
Double-wrapped bars	166.4	1670.5
Ribbed bars	172.1	1634.0

**Table 3:** Details of specimens.

Group	Grade of Concrete	Surface Type	*c*/*d*
1	C30	S	2
2	C40	D	2
3	C50	R	2
4	C50	D	4
5	C40	S	4
6	C30	R	4
7	C30	D	7
8	C40	R	7
9	C50	S	7

**Table 4:** Test results for pull-out specimens.

Specimen ID	Measured Bond Strength MPa	ACI Bond Strength MPa	Peak Slip (mm)	Failure Mode
S-50-7-1	9.82	14.78	4.52	P
S-50-7-2	9.02	14.78	3.13	P
S-50-7-3	11.21	14.78	3.95	P
D-50-4-1	15.27	14.27	8.24	P
D-50-4-2	18.31	14.27	10.91	P
D-50-4-3	16.15	14.27	6.2	S
R-50-2-1	12.51	13.93	2.28	P
R-50-2-2	11	13.93	1.6	P
R-50-2-3	13.76	13.93	3.87	P
S-40-4-1	10.4	13.01	11.67	P
S-40-4-2	14.82	13.01	18.1	P
S-40-4-3	13.37	13.01	13.27	P
D-40-2-1	14.03	12.70	6.18	S
D-40-2-2	13	12.70	5.79	S
D-40-2-3	13.71	12.70	7.7	S
R-40-7-1	16.88	13.48	6.65	P
R-40-7-2	12.72	13.48	2.82	P
R-40-7-3	16.71	13.48	7.63	P
S-30-2-1	8.26	11.11	3.26	S
S-30-2-2	9.02	11.11	4.7	S
S-30-2-3	9.67	11.11	7.92	P
D-30-7-1	10.85	11.79	12.83	P
D-30-7-2	9.21	11.79	14.5	P
D-30-7-3	13.43	11.79	11.59	P
R-30-4-1	8.82	11.38	1.98	P
R-30-4-2	6.95	11.38	1.12	P
R-30-4-3	9.42	11.38	1.96	P

**Table 5:** Material properties of SSSC cube and CFRP bar.

	Density (g/cm3)	Young’s Modulus (GPa)	Compressive Strength (MPa)	Tensile Strength (MPa)	Poison Ratio	Dilation Angle	Eccentricity	*f*_b0_/*f*_c0_	*K*	Viscosity Parameter
**SSSC (C50)**	2.42	308	58.9	3.72	0.2	30	0.1	1.16	0.667	0.001
**CFRP bar**	1.78	170	/	1700	0.3	/	/	/	/	/

**Table 6:** Parameters adopted in Figure 13.

Parameter	D-30-7-4	S-30-2-4	Parameter	D-40-7-2
F	4.788	4.363	a	0.72
G	−3.083	−2.682	γ	0.93
k	−0.168	−1.176	φ	0.07
			ω	1.33
			ρ	−1.17

## Data Availability

Not applicable.
